# N^6^-Methyladenosine (m^6^A) Methylation-Mediated Transcriptional Regulation in Maize Root Response to Salt Stress

**DOI:** 10.3390/plants15010036

**Published:** 2025-12-22

**Authors:** Wanling Ta, Zelong Zhuang, Jianwen Bian, Zhenping Ren, Xiaojia Hao, Lei Zhang, Yunling Peng

**Affiliations:** 1 College of Agronomy, Gansu Agricultural University, Lanzhou 730070, China; kellytwl@163.com (W.T.); zhuangzl3314@163.com (Z.Z.); bjwen1018@163.com (J.B.); renzp1003@163.com (Z.R.); haoxj772@163.com (X.H.); 18919106150@163.com (L.Z.); 2 Gansu Provincial Key Laboratory of Aridland Crop Science, Gansu Agricultural University, Lanzhou 730070, China

**Keywords:** salt stress, m^6^A methylation, transcriptional regulation, stress response, molecular mechanism

## Abstract

Salt stress represents a significant abiotic factor that constrains maize growth. Epigenetic modifications play a crucial role in enabling plants to respond effectively to such stresses. Among these alterations, m^6^A methylation, which is the most common post-transcriptional modification of eukaryotic mRNA, shows dynamic variations that are closely linked to stress responses. In this study, we conducted a transcriptome-wide m^6^A methylation analysis on maize roots from the inbred line PH4CV, following treatment with 180 mM NaCl. The results identified 1309 differentially m^6^A methylated peaks (DMPs) and 2761 differentially expressed genes (DEGs) under salt stress conditions. Association analysis revealed that 179 DEGs contain DMPs. Key pathways involved in stress responses, including Ca^2+^ signaling transduction and ABA signaling, as well as ion homeostasis regulation (involving AKT, HKT, and other families) and the reactive oxygen species scavenging system (including POD, SOD, and CAT), play crucial roles in coping with salt stress. Furthermore, we identified a total of 26 m^6^A-related genes, comprising 7 eraser genes, 10 reader genes, and 9 writer genes. Notably, several key salt-responsive genes, such as *RBOHB*, *AKT1*, *HKT1*, and *POD12*, are correlated with m^6^A modification. This study provides a comprehensive map of m^6^A methylation dynamics in maize roots under salt stress, laying a foundational resource for future investigations into the epigenetic regulation of salt tolerance in maize.

## 1. Introduction

Maize (*Zea mays* L.) ranks among the world’s three major cereal crops, alongside rice and wheat; however, it demonstrates moderate sensitivity to salt stress [1]. The problem of global soil salinization is growing more severe by the day. Data shows that more than 20% of farmland is already impacted, and this percentage is climbing at an annual rate of about 10% [2]. Predictions suggest that by 2050, up to 50% of the world’s arable land may face the threat of salinization, which could severely constrain food security and sustainable agricultural development [3]. High-salt environments inflict multiple damages on plants through osmotic stress, ion toxicity, and oxidative stress [4]. The buildup of excessive sodium (Na^+^) and chloride (Cl^−^) ions within cells disrupts the balance of ions and interferes with metabolic processes [5]. Additionally, it hampers seed germination [6], root development [7], and the growth of seedlings [8], which can ultimately result in reduced crop yield and quality. To manage salt stress, plants have developed intricate mechanisms for tolerance that include physiological and biochemical regulation, developmental adjustments, and structural modifications [9]. Calcium (Ca^2+^) signal transduction is crucial in the initial reaction to stress, transmitting signals to downstream defense systems through proteins such as CBL, CML, CaM, and CDPK [10]. During the absorption process, Na^+^ and K^+^ compete; under high-salt conditions, Na^+^ occupies K^+^ channels, adversely affecting K^+^ uptake. As an essential nutrient, K^+^ plays a role in a variety of physiological processes, such as regulating osmotic pressure, modulating enzymatic activity, and maintaining charge equilibrium [11]. Additionally, the NADPH oxidase family members RbohD and RbohF are activated under salt stress, mediating the rapid generation of reactive oxygen species (ROS). ROS serve not only as signaling molecules that modulate stress responses, but their excessive buildup can also result in oxidative harm [12,13]. To mitigate this, plants rely on the synergistic action of the antioxidant enzyme system to maintain ROS homeostasis: superoxide dismutase (SOD) converts O_2_^−^ into H_2_O_2_, while catalase (CAT) and peroxidase (POD) further decompose it into water and oxygen [14,15]. Consequently, it is essential to conduct a comprehensive investigation into the molecular processes through which maize reacts to salt stress to develop salt-resistant varieties and maintain both yield and quality.

RNA modifications exhibit a remarkable chemical diversity that greatly exceeds that of DNA modifications, with more than 150 unique types of RNA modifications recognized to date [16]. Different types of RNA methylation encompass mRNA, rRNA, tRNA, tmRNA, snRNA, miRNA, and viral RNA [17]. m^6^A is recognized as one of the most prevalent modifications found within the mRNA epitranscriptome [18]. m^6^A represents a dynamic and reversible modification mechanism, governed by methyltransferases (writers), demethylases (erasers), and proteins that recognize methylated sites (readers). This modification intricately modulates gene expression by affecting RNA stability, splicing, translation, and degradation [19]. In the model organism *Arabidopsis thaliana*, the writers identify several components: MTA, MTB, FIP37, VIRILIZER, FIONA1, and the E3 ubiquitin ligase HAKAI [20,21]. The eraser proteins include ALKBH9B and ALKBH10B [22], whereas the readers consist of ETC2, ETC3, ETC4, and CPSF30-L [23,24]. Within *Arabidopsis*, two main categories of typical m^6^A modification site sequences are recognized: one being RR[A]CH (R = G/A, H = A/C/U, [A] = m^6^A), and the other as URU[A]Y (Y = C/U) [25].

m^6^A exhibits significant biological functions in plants, playing a crucial role in their growth, development, and responses to environmental stresses through dynamic regulation [18]. Recent studies indicate that m^6^A not only participates in regulating several crucial developmental stages in plants, such as embryonic development [26], seed germination [27], flowering [20], and fruit ripening [28], but also impacts tissue formation and cell differentiation through its effects on the stability of mRNA and the efficiency of translation [29]. Additionally, the m^6^A modification has been extensively demonstrated to play a role in how plants respond to different abiotic and biotic stressors. In sorghum, the m^6^A methyltransferase *SbMTA*, along with the demethylase *SbALKBH10B*, has a notable influence on the plant’s ability to tolerate salt by modulating the stability of mRNA for genes related to the abscisic acid (ABA) signaling pathway [30]. Under drought stress, the distribution and abundance of m^6^A modifications in cotton undergo significant changes, particularly in drought-resistant varieties, where the m^6^A levels in the 5′UTR region increase, contributing to enhanced mRNA abundance of key genes and thereby improving drought resistance [31]. In tomatoes, low-temperature stress results in a reduction in overall m^6^A levels in anthers, which is accompanied by abnormal pollen development. The differentially modified transcripts are primarily enriched in pathways related to lipid metabolism, ATPase activity, and ATP binding [32]. Cadmium (Cd) stress inhibits root growth in plants, and key regulatory factors involved in the cadmium response, such as MAPK, WRKY, and MYB family genes, are positively regulated by m^6^A methylation [33]. Additionally, in terms of biotic stress, there is a substantial increase in the m^6^A modification level after maize is infected by the Maize chlorotic mottle virus (MCMV). Combined analyses of MeRIP-seq and RNA-seq demonstrated that m^6^A modification peaks are widely distributed in virus-infected plants and significantly alter the expression patterns of host genes [34]. As a crucial epitranscriptomic regulatory mechanism, m^6^A modification plays an extensive and sophisticated function in regulating plant growth and development, along with in responding to complex environmental stresses. It serves as a vital link between gene expression and environmental adaptation.

The m^6^A modification patterns in plants under salt stress exhibit significant species specificity and dependency on stress conditions. For instance, after treatment with 75 mM NaCl for 4 days, the differential m^6^A peaks in the roots of rice were predominantly enriched in the MAPK signaling pathway and in genes related to metal ion binding [35]. Under treatment with 200 mM NaCl, the genes exhibiting differential methylation in cotton roots were largely linked to various metabolic pathways, such as the synthesis of zeatin, metabolism of taurine and hypotaurine, regulation of ABC transporters, and synthesis of anthocyanins [36]. These differences suggest that m^6^A modification may mediate salt tolerance responses in various plants by regulating specific biological processes. As a C4 crop, maize may also utilize unique osmotic regulation and ion homeostasis mechanisms when coping with a high-salt environment. Therefore, deciphering the m^6^A modification map of maize under salt stress is of great significance for understanding its molecular mechanisms of salt tolerance. In this study, we used the maize inbred line PH4CV as the material and treated it with 180 mM NaCl. We systematically analyzed the physiological phenotypic changes and ion (Na^+^, K^+^, Ca^2+^, Cl^−^) accumulation characteristics of its root system. The MeRIP-seq method was utilized to perform a comprehensive assessment of the m^6^A methylome across the transcriptome. Additionally, an integrated analysis with transcriptomic data was carried out to uncover the essential role that m^6^A modification plays in controlling gene expression and its participation in salt tolerance in maize.

## 2. Results

### 2.1. Impact of Salt Stress on Maize Seedling Growth, Antioxidant Enzyme Activities, and Ion Contents

**Figure 1:**
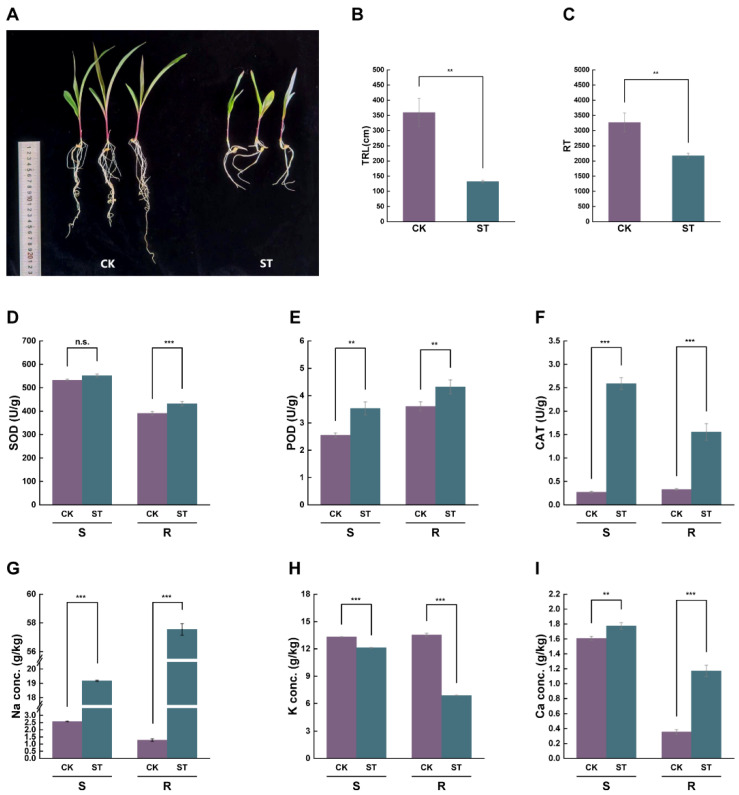
Impact of salt stress on the growth, antioxidant enzyme activities, and ion content of maize seedlings. CK: Control group (treated with distilled water); ST: salt-treated group (treated with 180 mM NaCl). S: Shoot; R: Root. (**A**) Phenotypic comparison of maize seedlings under CK and ST conditions. (**B**,**C**) The TRL and RT under two conditions. (**D**–**F**) Activities of antioxidant enzymes SOD, POD, and CAT in shoots and roots. (**G**–**I**) Na^+^, K^+^, and Ca^2+^ ion content in shoots and roots. All data are expressed as mean ± standard deviation from three replicates. The symbols ‘**’ and ‘***’ denote statistical significance for *p*-values of less than 0.01 and 0.001, respectively. ‘n.s.’ denotes ‘no significant difference’.

The growth of maize seedlings was markedly suppressed under salt stress (Figure 1A). In comparison to the control (CK) group, the total root length (TRL) and root tip number (RT) of the salt-treated (ST) group decreased by 63.3% and 33.5%, respectively (Figure 1B,C). In the roots, the activities of SOD, POD, and CAT increased significantly by 10.8%, 20.0%, and 37.3%, respectively. In contrast, the activity of SOD in the shoot showed no significant change, while the activities of POD and CAT increased significantly (Figure 1D–F). Meanwhile, the contents of Na^+^ and Ca^2+^ in both roots and shoots increased significantly, with Na^+^ and Ca^2+^ contents in the roots rising by 93.8% and 69.8%, respectively. Conversely, K^+^ content in both roots and shoots decreased significantly by 49.1% and 8.9%, respectively (Figure 1G–I). Additionally, Cl^−^ was significantly accumulated in both roots and shoots, with the Cl^−^ content in roots being notably higher than that in shoots (Supplementary Figure S1).

### 2.2. Genome-Wide Analysis of m^6^A Methylation Profile in Maize Roots Under Salt Stress

To investigate the response of maize roots to m^6^A methylation induced by salt stress, this study conducted MeRIP-seq analysis, resulting in a total of 867,634,514 original reads. After data filtering, the average proportion of high-quality bases (Q ≥ 20) in clean reads was 98.24%, the proportion of bases with Q ≥ 30 was 94.91%, and the GC content was between 46.67% and 52.54%. The proportions of adapters and low-quality reads were 0.65% and 0.39%, respectively (Supplementary Tables S1 and S2). PCA showed that the first two principal components (PC1 and PC2) explained 88.4% of the total variation (PC1: 63.8%, PC2: 24.6%), and the sample clustering was clear and reproducible (Supplementary Figure S2). The findings indicate that the sequencing data is high quality and satisfies the criteria for further analysis.

**Figure 2:**
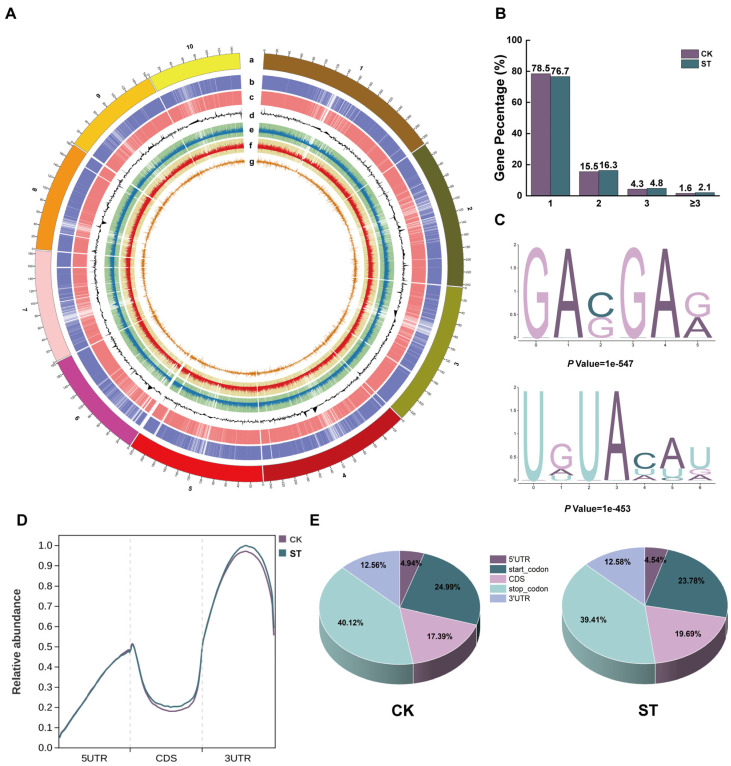
m^6^A methylation modification patterns in maize roots under salt stress. (**A**) Circos plot of m^6^A peaks and gene expression frequency of maize chromosomes (1–10) in CK and ST groups. (a) Chromosome distribution; (b) Heatmap of ST gene expression; (c) Heatmap of CK gene expression; (d) Bar chart of differentially expressed genes between ST and CK; (e) Heatmap and bar chart of ST m^6^A peak enrichment; (f) Heatmap and bar chart of CK m^6^A peak enrichment; (g) Bar chart of differentially m^6^A peaks between ST and CK. (**B**) Distribution of the number of m^6^A modification peaks in genes. (**C**) Dominant m^6^A sequence motifs identified in transcripts. (**D**) Genome-wide distribution density map of m^6^A peaks in different functional regions of transcripts (5′UTR, CDS, 3′UTR). (**E**) Proportional distribution of m^6^A peaks in various gene regions (5′UTR, start codon, CDS, stop codon, 3′UTR).

At the whole-genome level, we performed a systematic analysis of the distribution characteristics of m^6^A modifications. The Circos plot demonstrated that in the CK and ST groups, 37,627 and 39,616 m^6^A peaks were identified, respectively, indicating a widespread distribution across 10 chromosomes. Although the gene expression levels across each chromosome were relatively balanced, significant differences were observed in the density of m^6^A peaks: chromosome 1 exhibited the highest peak density, while chromosome 10 had the lowest (Figure 2A, Supplementary Figure S3). Further analysis of the m^6^A modification patterns on transcripts demonstrated that m^6^A peaks were predominantly enriched near the stop codon, a feature consistent in both groups. Under CK conditions, 78.5% of m^6^A-modified transcripts contained only one methylation peak, whereas this proportion was 76.7% in the salt treatment group; correspondingly, the proportion of transcripts with two or more m^6^A peaks was higher under salt stress (Figure 2B). Motif analysis using the MEME suite (v5.5.0) identified two conserved sequence motifs significantly enriched under salt stress conditions: G[A]VG[A]R ([A] = m^6^A, V = A/C/U, R = A/G) and UDU[A]HHD (D = A/G/U, H = A/C/U) (Figure 2C). After dividing the transcript into five functional regions, the analysis revealed that the m^6^A modification level within the CDS region of the ST group increased by 2.3% compared to the CK group (Figure 2D,E).

### 2.3. Identification and Functional Enrichment Analysis of DMPs in Maize Roots Under Salt Stress

**Figure 3:**
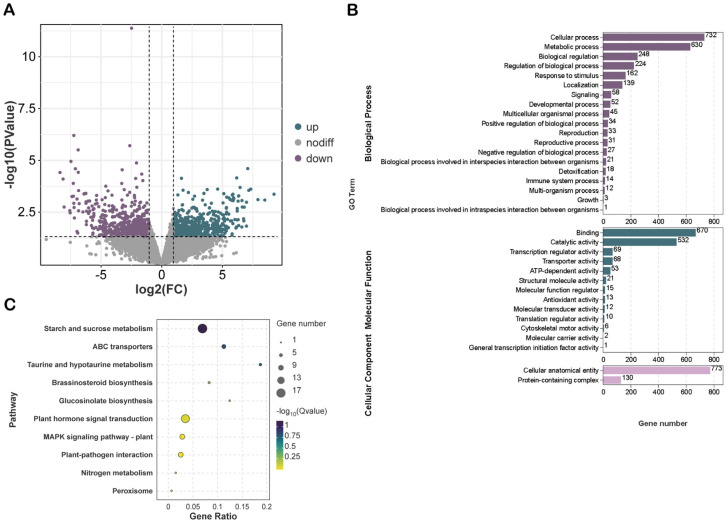
Functional enrichment analysis of DMPs under salt stress. (**A**) Volcano plot of DMPs. Green indicates upregulated peaks, purple indicates downregulated peaks, and gray indicates peaks with no differential expression. (**B**,**C**) GO and KEGG enrichment analysis of DMPs.

By comparing the CK group with the ST group, we identified a total of 1309 DMPs, comprising 581 DMPs with upregulated methylation levels and 728 DMPs with downregulated methylation levels (Figure 3A). GO enrichment analysis indicated that the genes associated with these DMPs were predominantly enriched in the biological process (BP) category, particularly for ‘cellular process,’ ‘metabolic process,’ and ‘developmental process.’ Furthermore, they were significantly enriched in the molecular function (MF) category for ‘binding,’ ‘catalytic activity,’ and ‘transporter activity,’ and mainly enriched in the cellular component (CC) category for ‘cellular anatomical entity’ (Figure 3B). Additionally, KEGG pathway analysis revealed that the genes related to these DMPs are primarily involved in key biological pathways, including ‘starch and sucrose metabolism,’ ‘plant hormone signal transduction,’ and ‘plant MAPK signaling pathway’ (Figure 3C).

### 2.4. Global Analysis and Functional Enrichment of DEGs in Maize Root Transcriptome Under Salt Stress

**Figure 4:**
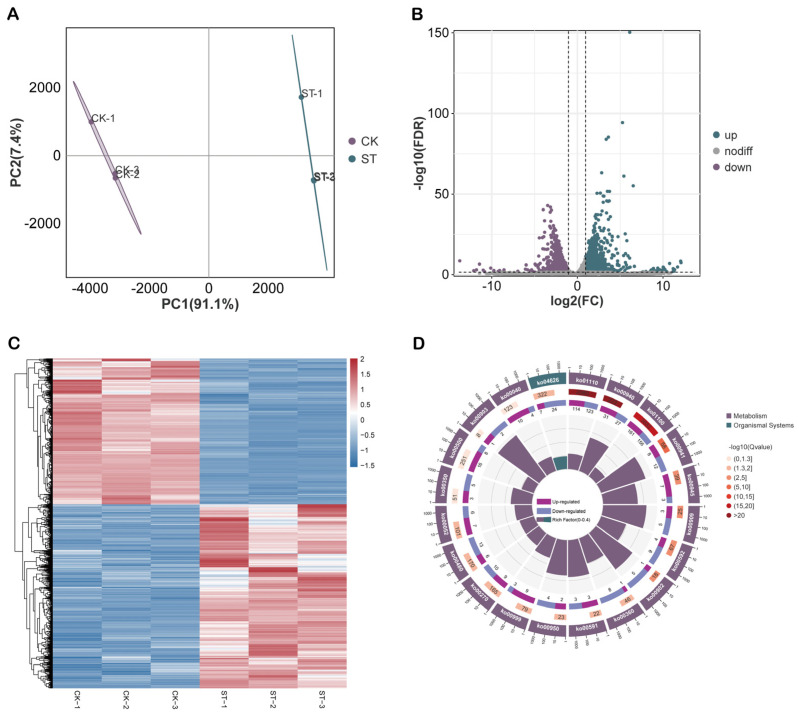
Global analysis and functional enrichment regulation of DEGs under salt stress (**A**) PCA analysis. (**B**) Volcano plot of DEGs. Green represents up-regulated genes, purple represents down-regulated genes, and gray represents non-differentially expressed genes. (**C**) Heatmap of all DEGs. (**D**) KEGG enrichment circle plot of the top 20 DEGs.

To investigate the effects of salt stress on gene expression within maize roots, this study performed RNA-Seq analysis, systematically examining the transcriptional variations between the CK and ST groups (Figure 4A). A total of 2761 DEGs were identified, of which 1539 were upregulated and 1222 were downregulated (Figure 4B). The clustering heatmap of DEGs exhibited strong consistency among the three biological replicates, with samples from the CK and ST groups distinctly clustered, indicating that salt stress significantly altered the transcriptional profile of maize roots (Figure 4C). KEGG pathway enrichment analysis revealed that both upregulated and downregulated DEGs were significantly enriched in multiple key pathways related to stress response and energy metabolism, with phenylpropanoid biosynthesis, flavonoid biosynthesis, and starch and sucrose metabolism being particularly prominent (Figure 4D).

### 2.5. Association Analysis of m^6^A Methylation and Transcriptome

**Figure 5:**
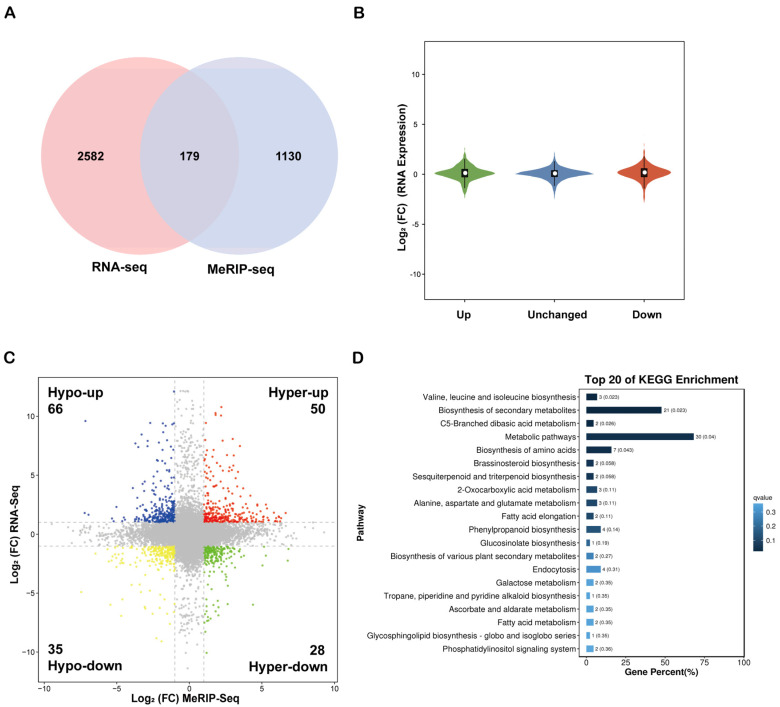
Integrated analysis of intergroup data from m^6^A-seq and RNA-seq. (**A**) Venn diagram of common differentially expressed genes between the two omics. (**B**) Violin plot of gene expression associated with peaks. (**C**) Four-quadrant plot illustrating the relationship between differential m^6^A methylation peaks and differentially expressed genes. Red dots represent Hyper-up, green dots indicate Hyper-down, yellow dots denote Hypo-down, and blue dots represent Hypo-up. (**D**) Bar chart of the top 20 enriched KEGG pathways for Hypo-up genes.

This study conducted an integrated analysis of MeRIP-seq and RNA-seq data to clarify the synergistic regulatory interaction between m^6^A methylation and gene expression. A total of 179 genes exhibited significant alterations in both methylation levels and expression quantities across the transcriptome and methylation datasets (Figure 5A). Notably, the changes in m^6^A modification levels (upregulation or downregulation) did not demonstrate a significant directional regulatory effect on the expression levels of the corresponding genes (Figure 5B). Specifically, 66 genes exhibited m^6^A hypomethylation coupled with upregulation (Hypo-up), 50 genes displayed m^6^A hypermethylation along with upregulation (Hyper-up), 35 genes manifested m^6^A hypomethylation associated with downregulation (Hypo-down), and 28 genes showed m^6^A hypermethylation linked to downregulation (Hyper-down) (Figure 5C). Furthermore, KEGG pathway enrichment analysis of the associated genes, particularly the top 20 most significantly upregulated genes in the Hypo-up category, indicated that these genes were predominantly enriched in pathways such as the ‘phosphatidylinositol signaling system,’ ‘ascorbate and aldarate metabolism,’ and ‘brassinosteroid biosynthesis’ (Figure 5D, Supplementary Figure S4).

### 2.6. Response of m^6^A Methylation-Related Gene Writers, Readers, and Erasers to Salt Stress

This study investigated the correlation between m^6^A methylation and salt stress-responsive genes in maize roots, identifying a total of 26 candidate genes related to RNA methylation, including 9 “writers,” 10 “readers,” and 7 “erasers.” Among these, the m^6^A methylation level of the eraser gene *ALKBH10B* (*Zm00001eb346760*) was significantly reduced (Diff. log_2_(FC) = −3.07). Among the readers, the m^6^A modification levels of *ECT2* (*Zm00001eb117310*) and *ECT3* (*Zm00001eb414030*) were downregulated (Diff. log_2_(FC) = −1.81 and −1.23), while the two *ECT4* genes (*Zm00001eb171010* and *Zm00001eb039740*) exhibited upregulation (Diff. log_2_(FC) = 1.24 and 1.30). Among the writers, the m^6^A level of *FIP37* (*Zm00001eb231610*) significantly increased (Diff. log_2_(FC) = 1.69), whereas those of *MTB1* (*Zm00001eb339360*) and *MTB2* (*Zm00001eb155410*) significantly decreased (Diff. log_2_(FC) = −2.02 and −1.42, respectively). Notably, although the m^6^A modification status of the aforementioned genes was altered, their transcriptional expression levels did not reach significant differences. The only exception was the *ECT3* gene (*Zm00001eb156740*), whose m^6^A methylation level was downregulated (Diff. log_2_(FC) = −1.19), while its gene expression level was upregulated (log_2_(FC) = 1.03), indicating a response to salt stress at both the methylation and expression levels (Table 1).

### 2.7. m^6^A Modification Characteristics and Expression Validation of Key Salt-Tolerance Related Genes

**Figure 6:**
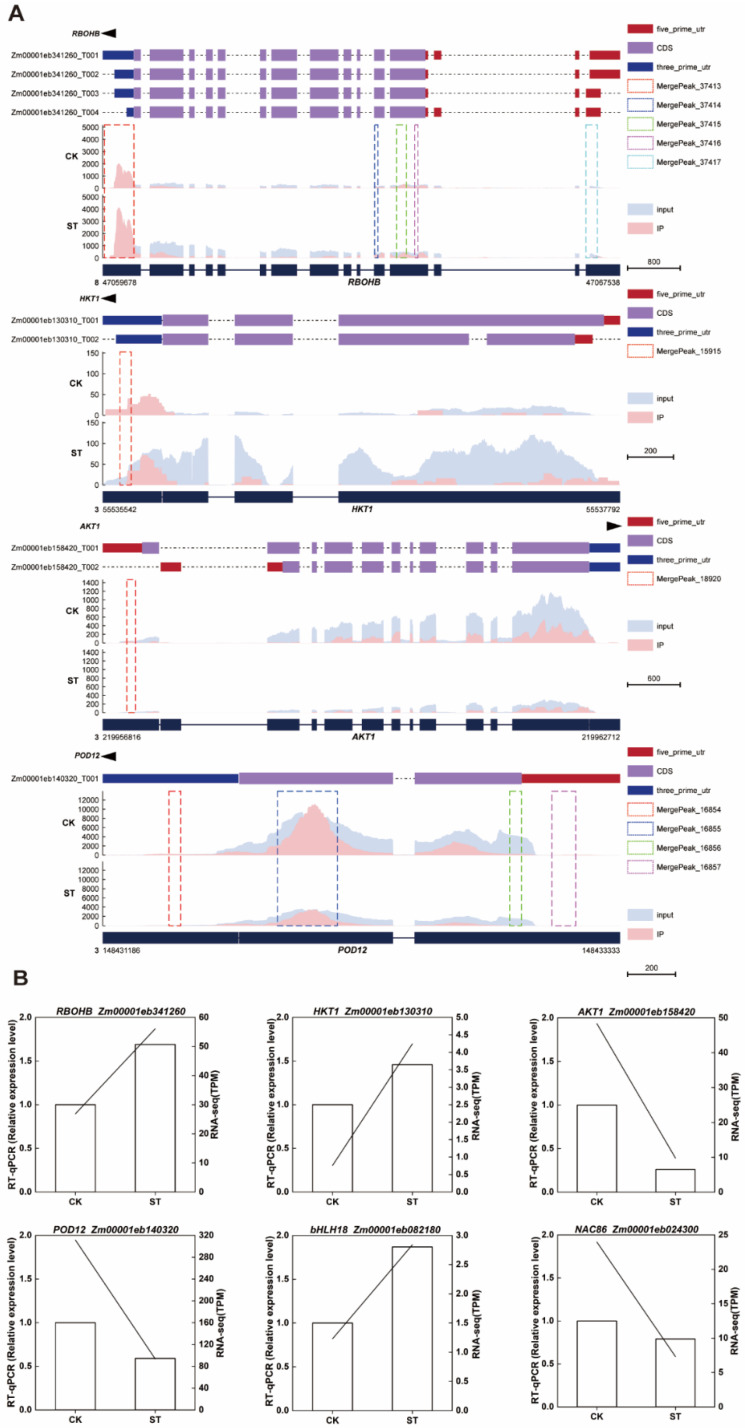
m^6^A methylation profile and gene expression in maize under salt stress. (**A**) IGV tracks show the distribution of m^6^A immunoprecipitation enrichment signals (IP) and transcript abundance (Input) of *RBOHB*, *HKT1*, *AKT1*, and *POD12* on the genome under CK and salt treatment conditions. The dashed boxes indicate m^6^A peaks. (**B**) The left bar graph displays the relative expression levels determined by qRT-PCR; the right line graph shows the corresponding gene expression levels from RNA-seq.

Through the visualization analysis of MeRIP-seq data using the Integrative Genomics Viewer (IGV), we identified significant m^6^A enrichment peaks on multiple key genes associated with salt tolerance in the maize genome. *RBOHB* (*Zm00001eb341260*), located on chromosome 8, exhibited strong m^6^A enrichment signals in the 5′UTR, CDS, and 3′UTR regions of its transcripts (T001-T004). Two important ion transport-related genes, *HKT1* (*Zm00001eb130310*) and *AKT1* (*Zm00001eb158420*), are both located on chromosome 3. The m^6^A peak of *HKT1* is primarily concentrated in the 3′UTR region, while the peak of *AKT1* is positioned in the 5′UTR region. Additionally, the peroxidase gene *POD12* (*Zm00001eb140320*) exhibits significant methylation enrichment in the CDS region of chromosome 3 (Figure 6A). To verify the reliability of the transcriptome data, we employed RT-qPCR to detect the expression levels of the aforementioned four genes. The results indicated that the expression trends obtained by RT-qPCR were highly consistent with the RNA-seq data, further confirming the reliability of the expression changes in *RBOHB*, *HKT1*, *AKT1*, *POD12*, *bHLH18* and *NAC86* under salt stress (Figure 6B).

## 3. Discussion

Salt stress is a significant abiotic factor that hinders plant growth and agricultural yield, mainly by causing osmotic pressure, ion toxicity, and oxidative damage [4]. To alleviate these negative impacts, plants have developed a variety of adaptive mechanisms, such as enhancing antioxidant enzyme activity and regulating hormone signaling, to maintain the balance of ROS and ion homeostasis [37]. This research demonstrated that the application of salt treatment significantly suppressed the growth of maize seedlings, which was indicated by a notable decrease in the TRL and RT, aligning with previous research results [38]. This suggests that salt stress exerts a profound inhibitory effect on root development. As the primary organ for detecting soil salinity, impaired root growth directly impacts the absorption of water and nutrients, thereby influencing the overall growth performance of the plant. SOD, POD, and CAT are crucial enzymes involved in ROS scavenging. Our results indicate that salt treatment significantly elevated the activities of these three enzymes in maize roots, demonstrating that the antioxidant defense system was effectively activated to counteract oxidative damage induced by salt stress. Additionally, salt stress resulted in a substantial buildup of Na^+^ in the roots, disrupting cellular ion balance. Because of the structural resemblances between Na^+^ and K^+^, excessive Na^+^ accumulation in the cytoplasm competitively inhibits the uptake and function of K^+^, consequently interfering with the activities of enzymes that rely on K^+^ for activation. Since Na^+^ cannot fulfill the physiological roles of K^+^, this leads to severe ion toxicity [39]. The findings of this study reveal that salt treatment significantly increased Na^+^ and Ca^2+^ content in the roots and shoots of maize, while K^+^ content notably decreased, with these changes being particularly pronounced in the roots. This further substantiates the disruptive effect of salt stress on ion homeostasis.

In recent years, the regulatory role of RNA epigenetic modifications, particularly m^6^A methylation, in plant responses to abiotic stress has garnered increasing attention [40]. Therefore, a thorough examination of the dynamic alterations in m^6^A modifications under salt stress is crucial for understanding the mechanisms of salt tolerance in maize from the perspective of post-transcriptional regulation. The analysis of the chromosomal distribution of m^6^A peaks revealed that, although the total number of m^6^A peaks did not exhibit significant changes under salt stress, there were notable differences in their distribution across different chromosomes: chromosome 1 displayed the highest density, whereas chromosome 10 exhibited the lowest. This finding aligns with previous studies that indicated an uneven distribution of m^6^A in maize leaves [41]. Prior research has generally suggested that m^6^A modifications are predominantly enriched near the 3′UTR and the stop codon [42]. In this study, significant enrichment in the termination codon region was observed not only in the roots of maize seedlings but also in the initiation codon region, suggesting that the distribution pattern of m^6^A may vary across different plant tissues or developmental stages. Notably, the proportion of m^6^A modification in the CDS region increased by 2.30% under salt stress contrasted with the CK group. Previous studies have demonstrated that m^6^A enrichment in the CDS region is typically associated with enhanced mRNA stability and increased translation efficiency. For instance, in poplar, transcripts with hypermethylated m^6^A in the CDS region often exhibit higher expression levels [43]. Furthermore, the proportion of transcripts containing two or more m^6^A peaks significantly increased under salt stress. This multimodal modification may enhance plant adaptability to stress by increasing mRNA stability or translation efficiency [44,45]. Regarding sequence characteristics, it is known that m^6^A methyltransferases in plants can recognize the UGUA sequence, and the reader protein ECT2 specifically binds to the URUAY motif [24]. In this study, the “G[A]VG[A]R ([A] = m^6^A, V = A/C/U, R = A/G)” motif shares the core features of the canonical plant RRACH motif (R = G/A, H = A/C/U). Similarly, the “UDU[A]HHD (D = A/G/U)” motif is a variant of the known URU[A]Y (Y = C/U) motif. This finding confirms the evolutionary conservation of m^6^A motifs in plants while also suggesting a degree of sequence preference or context-specific variation under salt stress in maize [41].

Salt stress may cause a deficiency in water or osmotic pressure in plants, which in turn can trigger the closure of stomata and harm photosynthetic pigments [46]. Plants mitigate cellular damage through various mechanisms, including the regulation of ion homeostasis, osmotic balance, and the antioxidant system, in response to salt stress [47]. This study identified 1309 DMPs and 2761 DEGs, whose functions are significantly enriched in key biological processes such as Ca^2+^ signaling transduction, ABA signaling pathways, ion transport, and ROS scavenging. During the initial phase of salt stress, the rhizosphere salt concentration rapidly increases, triggering osmotic stress, activating hyperosmosensors, and initiating Ca^2+^ signaling [10]. This study found that Ca^2+^ enters the cytoplasm through the cyclic nucleotide-gated channel *CNGC4*, activates the downstream calcium-dependent protein kinase *CDPK20*, and transmits the signal downstream through the calmodulin-binding protein *CBP*, involving proteins or transcription factors participating in cellular defense. Meanwhile, the ABA signaling pathway is activated. After ABA accumulation, it is sensed by the receptor *PYL10*, which then inhibits the protein phosphatase *PP2C37*, relieving its suppression of SnRK2-type protein kinases. Upon activation, *SnRK2.9* can phosphorylate transcription factors such as bZIP, thereby regulating the expression of downstream stress-tolerance genes [48]. Additionally, salt stress induces the accumulation of ROS, thereby activating the MAPK signaling pathway. For example, *MPK3* and *MPK6* are significantly activated under salt stress and are involved in regulating the oxidative stress response. These kinases modulate ROS homeostasis and the activity of antioxidant enzymes by phosphorylating downstream target proteins, thereby alleviating the damage caused by salt stress to plants [49]. Ultimately, these signaling pathways ultimately converge on the regulation of a series of key transcription factors, including members of the WRKYs (*Zm00001eb199530*, *Zm00001eb149300*, *Zm00001eb203940* and *Zm00001eb169460*), ARFs (*Zm00001eb157270*, *Zm00001eb082150* and *Zm00001eb067270*), NACs (*Zm00001eb176840*, *Zm00001eb320820* and *Zm00001eb024300*) and bHLHs (*Zm00001eb082180* and *Zm00001eb011370*), which play a central role in regulating the expression of salt tolerance-related genes, collectively constructing the molecular regulatory network for plants to cope with salt stress.

During salt stress, various channels, transporters, and antiporters play roles in maintaining sodium/potassium homeostasis [50]. In terms of ion balance regulation, the vacuolar H^+^/Ca^2+^ antiporter *CAX3* and the sodium/hydrogen exchanger *NHX4* contribute to Ca^2+^ compartmentalization and Na^+^/K^+^ homeostasis maintenance; the plasma membrane H^+^-ATPase *AHA9* affects the proton gradient, regulating Na^+^ efflux; while the vacuolar H^+^-pyrophosphatase *AVP5* enhances proton pump activity, providing the driving force for Na^+^ compartmentalization. Furthermore, the low-affinity Hypo-down gene potassium channel *AKT1* (Diff. log_2_(FC) = −5.55, log_2_(FC) = −2.31) is a major contributor to K^+^ uptake and transport; the Hypo-up gene sodium transporter *HKT1* (Diff. log_2_(FC) = −3.20, log_2_(FC) = 2.48) mediates Na^+^ uptake in maize. In the oxidative stress response, Ca^2+^ binds to the hyper-up gene NADPH oxidase *RBOHB* (Diff. log_2_(FC) = 6.06, log_2_(FC) = 1.06), promoting ROS generation; while antioxidant enzymes such as SOD, CAT, and POD are induced to express, synergistically scavenging excess ROS and maintaining redox homeostasis. Studies have shown that scavenging ROS or enhancing antioxidant capacity can significantly improve plant salt tolerance [51].

**Figure 7:**
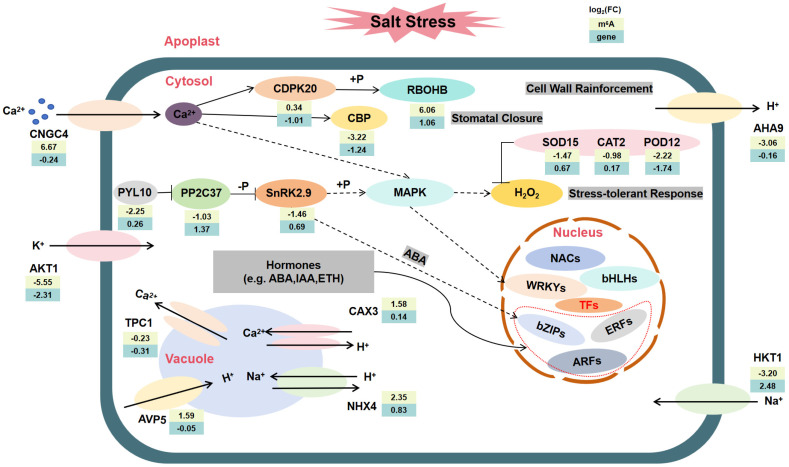
A working model of the m^6^A transcriptome regulatory network in maize roots under salt stress. Under salt stress, the molecular processes mediated by both DMPs and DEGs are involved in the biological processes between the cytoplasm, nucleus, and vacuoles in maize root cells.

In summary, this study presents a comprehensive model (Figure 7, Supplementary Table S3) that elucidates the role of m^6^A modification in the response of maize roots to salt stress. The integrated analysis of transcriptome and m^6^A methylation indicates that m^6^A modification may coordinate the regulation of Ca^2+^ signaling, the ABA pathway, ion transport, and antioxidant defense at multiple levels, thereby demonstrating its multidimensional regulatory function in the adaptation to abiotic stress.

## 4. Materials and Methods

### 4.1. Experimental Materials and Design

The salt-sensitive maize inbred line PH4CV served as the experimental material for this research. Uniform seeds were selected and subjected to surface sterilization using a 0.5% NaClO solution for 30 min. Subsequently, the seeds were rinsed with distilled water 3 to 5 times and then allowed to soak in distilled water for 12 h. We then sowed five seeds in each pot (15 cm × 12 cm × 10 cm) filled with vermiculite that had been pre-mixed with a 180 mM NaCl solution. For the CK group, distilled water was utilized in place of the salt solution while maintaining all other conditions identical. Plants were grown in a greenhouse where daytime temperatures were maintained at 25 °C and nighttime temperatures at 22 °C, under a light regimen of 14 h of illumination and 10 h of darkness. Every two days, they received 50 mL of either the 180 mM NaCl solution (ST) or distilled water (CK). Each experimental condition included three biological replicates. After a growth period of 14 days, when the plants developed to the three-leaf and one-heart stages, root samples were gathered, quickly flash-frozen in liquid nitrogen, and preserved at −80 °C for future RNA extraction and sequencing [52].

### 4.2. Measurement of Growth and Physiological Parameters

Root and shoot samples of maize seedlings were collected from both the CK and ST groups. Root morphology was analyzed by scanning the root samples with a root scanner (Epson Expression 12000XL; Epson America, Inc., Los Alamitos, CA, USA) at a 600 dpi and using the WinRHIZO system (v 2022a) to determine TRL and RT number. For antioxidant enzyme activity assays, SOD activity was measured at 600 nm using the nitroblue tetrazolium (NBT) reduction method; POD activity was determined at 470 nm via the guaiacol oxidation method; and CAT activity was assessed at 240 nm by monitoring the decomposition of hydrogen peroxide (H_2_O_2_) [53].

### 4.3. Ion Concentration Determination

The root samples, once frozen, were dried at 80 °C for a duration of 48 h before being ground into a fine powder. Following this, the powdered samples were digested using a microwave digestion system with 70% nitric acid (HNO_3_) at a temperature of 120 °C for 4 h. Atomic absorption spectroscopy was utilized to determine the concentrations of Na^+^, K^+^, and Ca^2+^.

An extra 0.5 g of the dried sample was subjected to extraction with 50 mL of deionized water at 80 °C for 1 h using a water bath. The Cl^−^ concentration was assessed by potentiometric titration with a 0.01 M AgNO_3_ solution, recording both the volume of AgNO_3_ used and the corresponding potential readings. The analysis incorporated three biological replicates for each treatment, along with three technical replicates for every biological sample. Blank controls were conducted simultaneously to guarantee measurement precision.

### 4.4. RNA Extraction, Library Construction, and Sequencing

Total RNA extraction was performed utilizing TRIzol reagent (Invitrogen, Carlsbad, CA, USA). The integrity and quality of RNA were evaluated using an Agilent 2100 Bioanalyzer (Agilent Technologies, Santa Clara, CA, USA) and further confirmed through RNase-free agarose gel electrophoresis. The assessment of RNA quality has been included in Supplementary Table S4. Enrichment of Poly(A)+ mRNA from the total RNA was carried out with Oligo(dT) magnetic beads. Subsequently, the purified mRNA was fragmented into segments of approximately 100 nucleotides by incubation in a fragmentation buffer (10 mM Tris-HCl, pH 7.0, 10 mM ZnCl_2_) at 94 °C for a duration of 5–7 min. The fragmented RNA was then split into two aliquots: one reserved as the input control and the other processed for m^6^A immunoprecipitation (m^6^A-IP). For the m^6^A-IP procedure, RNA fragments were incubated with a specific anti-m^6^A antibody (Synaptic Systems, Göttingen, Germany; Cat. No. 202003). The fragments containing m^6^A that were bound to the antibody were subjected to immunoprecipitation, elution, and then reverse transcription into cDNA using random primers with the NEBNext Ultra RNA Library Prep Kit for Illumina (New England Biolabs, Ipswich, MA, USA; #7530). The cDNA fragments produced underwent end repair, adenylation, and ligation with Illumina sequencing adapters to create the final libraries. Following quality control, the libraries were sequenced on an Illumina HiSeq 4000 platform ((Illumina Inc., San Diego, CA, USA) at Gene Denovo Biotechnology Co. (Guangzhou, China)).

### 4.5. Bioinformatic Analysis of MeRIP-Seq Data

Raw reads were initially processed to eliminate adapter sequences and low-quality bases. Contamination from rRNA was eliminated by mapping the reads to an rRNA reference database through the use of Bowtie2 (v2.2.8) [54]. The remaining non-rRNA reads were subsequently mapped to the maize reference genome (B73 RefGen_v4). MACS2 (v2.1.2) [55] was utilized to identify m^6^A-enriched peaks, employing a dynamic Poisson model to calculate peak significance (*p*-value), followed by false discovery rate (FDR) correction (FDR < 0.05). To ensure the reliability of the peaks, only those detected in ≥50% of biological replicates were retained. The peaks were annotated to the 5′UTR, CDS, 3′UTR, and other functional regions. The DREME module of the MEME suite (v5.5.0; http://meme-suite.org/, accessed on 5 July 2024) was employed to identify enriched sequence motifs within the m^6^A peaks. DiffBind (v2.8) [56] was utilized to merge peaks across samples and calculate RPM (Reads Per Million mapped reads). DMPs were defined as those with FDR < 0.05 and |log_2_FC)| ≥ 1.

### 4.6. Bioinformatics Analysis of RNA-Seq Data

Following the acquisition of raw sequencing data containing adapter sequences or low-quality bases, initial quality control was conducted using fastp (v0.18.0) [57] to filter reads and remove adapters, resulting in high-quality clean reads. The clean reads were subsequently aligned against an rRNA database using Bowtie2 (v2.2.8) [54] to eliminate rRNA-matching reads, thereby reducing interference from highly abundant non-coding RNAs. Subsequently, an index of the maize reference genome was created using HISAT2 (v2.1.0) [58], which also aligned the paired-end clean reads to this reference genome, applying default settings. Following the alignment process, transcript assembly for each sample was carried out with StringTie (v1.3.1) [59,60], relying on the annotations from the reference genome. Gene expression quantification was carried out using RSEM [61] to calculate FPKM values for each transcript, allowing for the evaluation of gene expression abundance and variation. Inter-sample correlation analysis and PCA were conducted using R (v4.3.1) with the gmodels package (http://www.r-project.org/, accessed on 12 July 2024) to assess replicate consistency and inter-group differences. DEGs analysis was cross-validated using both DESeq2 [62] and edgeR [63] algorithms. DEGs were identified using thresholds of FDR < 0.05 and |log_2_(FC)| ≥ 1. The final set of DEGs was subsequently utilized for functional enrichment analysis and correlation studies with m^6^A modification patterns.

### 4.7. GO and KEGG Enrichment Analysis

GO [64] enrichment analysis was conducted to identify significantly enriched biological terms among DEGs and DMPs. The GO database consists of three ontologies: Molecular Function, Cellular Component, and Biological Process. All candidate genes were mapped to their corresponding GO terms (http://www.geneontology.org/, accessed on 12 July 2024), followed by hypergeometric testing to evaluate the significance of enrichment. Terms with an FDR < 0.05, after multiple testing correction, were deemed statistically significant. For pathway analysis, genes were annotated against the KEGG [65] database to identify significantly enriched metabolic pathways and signaling cascades. The significance of enrichment was similarly assessed using hypergeometric tests with FDR < 0.05. The most significantly enriched GO terms and KEGG pathways were selected for downstream interpretation of biological functions and regulatory networks.

### 4.8. qRT-PCR Validation

Total RNA was extracted using TRIzol reagent (Invitrogen, Carlsbad, CA, USA) and reverse transcribed into complementary DNA (cDNA) using SPARKscript™ II All-in-one RT SuperMix (Sangon Biotech, Shanghai, China). Quantitative real-time PCR (qRT-PCR) was conducted on a QuantStudio 5 Real-Time PCR System (Thermo Scientific, Waltham, MA, USA) utilizing 2× Universal SYBR Green qPCR Mix. Gene-specific primers, the sequences of which are provided in Supplementary Table S5, were designed using Primer Premier 5.0 software. Each sample was analyzed in triplicate, with three biological replicates, each consisting of technical replicates. Relative gene expression levels were calculated using the 2^−ΔΔCT^ method, with Actin serving as the internal reference gene for normalization.

### 4.9. Statistical Analysis

Statistical analyses were carried out employing IBM SPSS Statistics version 26.0. The differences between groups were evaluated using two-tailed Student’s *t*-tests. Data visualization was executed utilizing Origin 2021. To guarantee the reliability and reproducibility of the results, all experiments were independently repeated a minimum of three times. The data is presented as mean ± standard error (SE), with statistical significance established at *p* < 0.05.

## 5. Conclusions

This study elucidates the dynamic modification of m^6^A methylation and its association with salt-responsive genes in maize roots under salt stress by integrating MeRIP-seq and RNA-seq methodologies. Under salt stress conditions, we identified 1309 DMPs and 2761 DEGs. Further association analysis revealed that 179 DEGs contained DMPs. Key stress response pathways, including Ca^2+^ signaling transduction and ABA signaling, as well as mechanisms for ion homeostasis regulation (such as AKT and HKT families) and ROS scavenging systems (including POD, SOD, and CAT), play significant roles in coping with salt stress. Notably, multiple key salt-responsive genes, including *RBOHB*, *AKT1*, *HKT1*, and *POD12*, showed differential m^6^A methylation. This study provides insights into the m^6^A epitranscriptome associated with salt stress in maize, laying a foundational resource for future investigations into the epigenetic regulation of salt tolerance in maize.

## Figures and Tables

**Table 1:** RNA methylation-related genes in maize.

Gene_Id	Symbol	Type	m^6^A			Gene_Expression		
			Diff. log_2_ (FC)	*p*-Value	Up/Down	log_2_ (FC)	*p*-Value	Up/Down
*Zm00001eb346760*	ALKBH10B	eraser	−3.07	0.088	Down	−0.17	0.477	NC
*Zm00001eb287600*	ALKBH10B	eraser	−0.92	0.170	NC	−0.07	0.164	NC
*Zm00001eb010150*	ALKBH10B	eraser	−0.44	0.493	NC	0.45	0.000 **	NC
*Zm00001eb380830*	ALKBH2	eraser	−0.36	0.552	NC	0.92	0.045 *	NC
*Zm00001eb047810*	ALKBH6	eraser	−0.29	0.552	NC	−0.14	0.486	NC
*Zm00001eb071740*	ALKBH8	eraser	−0.36	0.475	NC	0.15	0.062	NC
*Zm00001eb377750*	ALKBH9B	eraser	−0.70	0.115	NC	0.45	0.000 **	NC
*Zm00001eb403260*	ECT2	reader	0.16	0.733	NC	0.30	0.001 **	NC
*Zm00001eb360390*	ECT2	reader	0.11	0.847	NC	0.71	0.000 **	NC
*Zm00001eb117310*	ECT2	reader	−1.81	0.217	Down	−0.74	0.843	NC
*Zm00001eb057150*	ECT2	reader	0.29	0.355	NC	0.01	0.154	NC
*Zm00001eb414030*	ECT3	reader	−1.23	0.090	Down	−0.21	0.566	NC
*Zm00001eb156740*	ECT3	reader	−1.19	0.077	Down	1.03	0.000 **	Up
*Zm00001eb300970*	ECT4	reader	0.54	0.235	NC	0.58	0.000 **	NC
*Zm00001eb171010*	ECT4	reader	1.24	0.086	Up	−0.14	0.297	NC
*Zm00001eb117080*	ECT4	reader	0.53	0.235	NC	0.60	0.000 **	NC
*Zm00001eb039740*	ECT4	reader	1.30	0.037 *	Up	−0.03	0.152	NC
*Zm00001eb231610*	FIP37	writer	1.69	0.002 **	Up	−0.07	0.266	NC
*Zm00001eb209200*	FIP37	writer	−0.39	0.320	NC	0.15	0.038 *	NC
*Zm00001eb221140*	HAKAI	writer	0.33	0.449	NC	0.50	0.000 **	NC
*Zm00001eb044800*	HAKAI	writer	−0.27	0.494	NC	0.15	0.008 **	NC
*Zm00001eb003840*	MTB	writer	−0.13	0.790	NC	0.30	0.011 *	NC
*Zm00001eb339360*	MTB1	writer	−2.02	0.087	Down	−0.01	0.134	NC
*Zm00001eb119000*	MTB1	writer	0.46	0.253	NC	0.08	0.049 *	NC
*Zm00001eb155410*	MTB2	writer	−1.42	0.011 *	Down	−0.69	0.087	NC
*Zm00001eb220020*	VIR	writer	−0.34	0.472	NC	−0.21	0.863	NC

Note: ‘Up’ indicates up-regulation; ‘Down’ indicates down-regulation; ‘NC’ indicates no change. ‘*’ means *p*-Value < 0.05, significant; ‘**’ means *p*-Value < 0.01, extremely significant.

## Data Availability

The original contributions presented in the study are included in the article/Supplementary Materials. Further inquiries can be directed to the corresponding authors.

## Supplementary Materials

The following supporting information can be downloaded at: https://www.mdpi.com/article/10.3390/plants15010036/s1, Supplementary Table S1: Reads Filtering Information Statistics. Supplementary Table S2: Base quality analysis. Supplementary Table S3: Differential gene expression and methylation profiles of maize roots under salt stress. Supplementary Table S4: m^6^A-IP/input RNA quality inspection. Supplementary Table S5: List of primer pairs used in qRT-PCR. Supplementary Figure S1: The content of Cl^−^ was determined in the stems and roots of seedlings, respectively. Supplementary Figure S2: Principal component analysis of control and salt-treated samples. Supplementary Figure S3: Peak distribution on the genomes of different chromosomes. Supplementary Figure S4: The enrichment significance of m^6^A modified differential genes at the level of KEGG pathway categories (q value).
