# Supplementary material for: N6-Methyladenosine (m6A) Methylation-Mediated Transcriptional Regulation in Maize Root Response to Salt Stress
Source: Plants (Basel). 2025 Dec 22;15(1):36. doi: 10.3390/plants15010036 (PMC12787851; doi:10.3390/plants15010036)
Supplement: Supplementary file 1 [file plants-15-00036-s001.zip › Supplementary Figure.pdf]

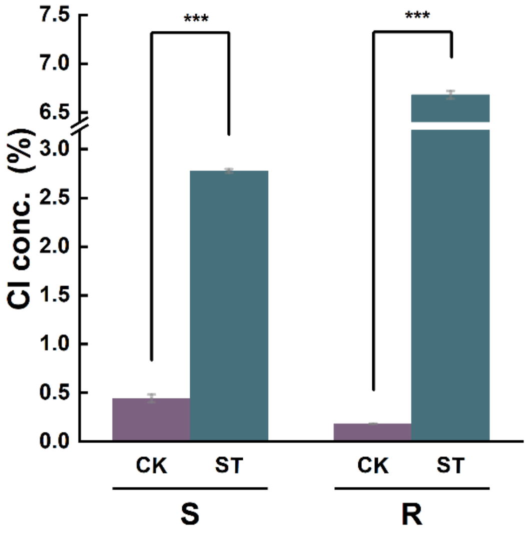

Supplementary Figure S1. The content of Cl^- was determined in the stems and roots of seedlings, respectively

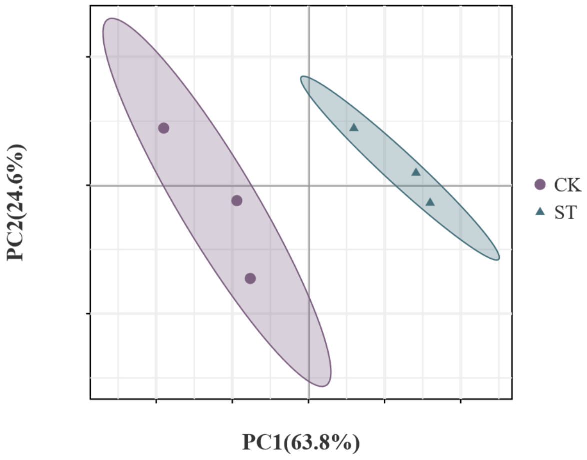

Supplementary Figure S2. Principal component analysis of control and salt-treated samples

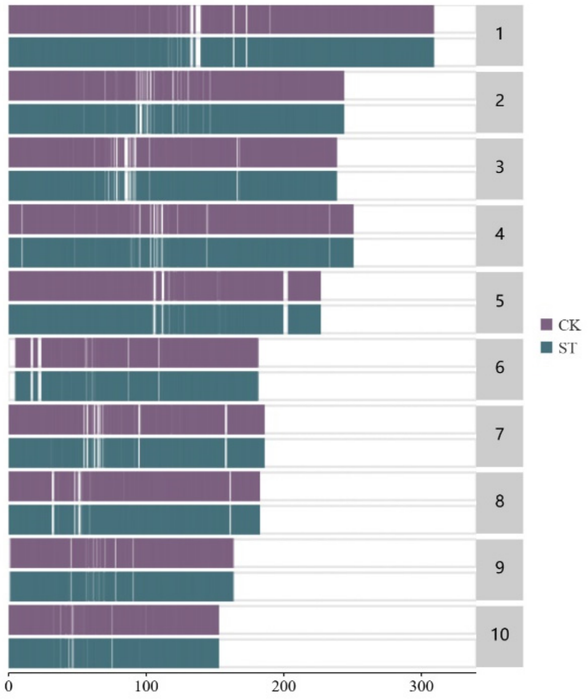

Supplementary Figure S3. Peak distribution on the genomes of different chromosomes

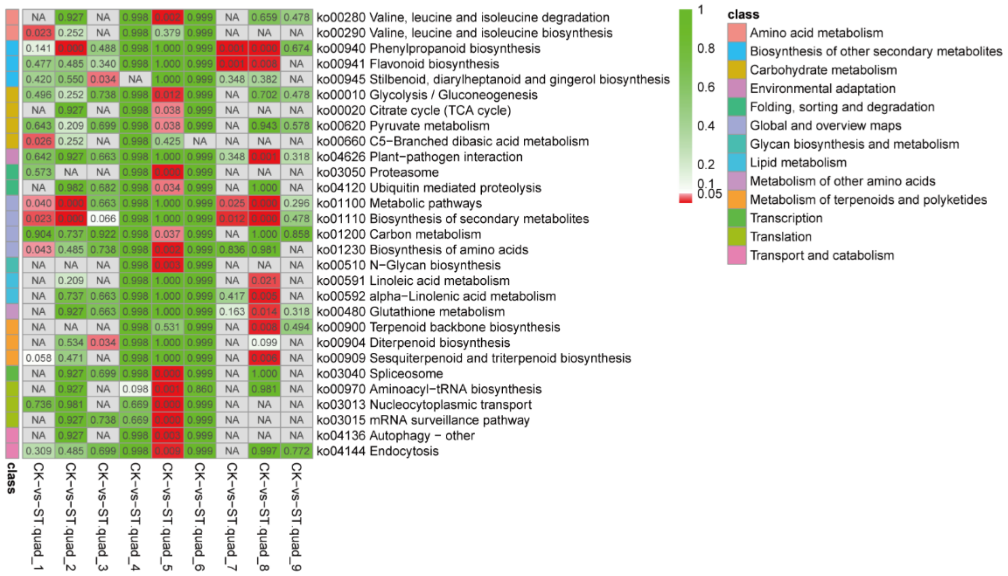

Supplementary Figure S4. The enrichment significance of m6 A modified differential genes at the level of KEGG pathway categories (q value)
