# Supplementary material for: N6-Methyladenosine (m6A) Methylation-Mediated Transcriptional Regulation in Maize Root Response to Salt Stress
Source: Plants (Basel). 2025 Dec 22;15(1):36. doi: 10.3390/plants15010036 (PMC12787851; doi:10.3390/plants15010036)
Supplement: Supplementary file 1 [file plants-15-00036-s001.zip › Supplementary Table.pdf]

Supplementary Table S1. Reads filtering information statistics

Sample	Raw Data	Clean Data (%)	Adapter (%)	Low Quality (%)
CK-IP-1	87098014	98.16	1.51	0.34
CK-IP-2	82355226	98.23	1.42	0.35
CK-IP-3	87611292	98.00	1.46	0.54
CK-input-1	43884286	99.36	0.05	0.59
CK-input-2	58137072	99.70	0.05	0.25
CK-input-3	56205718	99.74	0.05	0.22
ST-IP-1	86986062	98.33	1.27	0.39
ST-IP-2	94380096	97.87	1.78	0.34
ST-IP-3	84015184	99.14	0.11	0.75
ST-input-1	70301214	99.55	0.05	0.41
ST-input-2	58539624	99.71	0.05	0.25
ST-input-3	58120726	99.71	0.05	0.24

Supplementary Table S2. Base quality analysis

Sample	Clean Data (bp)	AF_Q20 (%)	AF_Q30 (%)	AF_GC (%)
CK-IP-1	9264797714	98.70	96.24	48.56
CK-IP-2	9169502396	98.95	96.93	46.83
CK-IP-3	9435784505	98.53	95.83	47.87
CK-input-1	6474209370	98.18	94.85	52.54
CK-input-2	8661313923	97.42	92.62	52.08
CK-input-3	8370663423	97.66	93.05	52.10
ST-IP-1	9305586938	98.63	96.02	47.10
ST-IP-2	10012639675	98.96	96.98	47.29
ST-IP-3	9505628323	98.19	94.63	46.67
ST-input-1	10402951442	98.66	96.04	52.26
ST-input-2	8717138473	97.45	92.70	51.97
ST-input-3	8660086599	97.60	93.05	51.80

Supplementary Table S3. Differential gene expression and methylation profiles of maize roots under salt stress

Gene_Id	Symbol	Description	m^6A		Gene_expression	
			Diff. log_2(FC)	Up/Down	log_2(FC)	Up/Down
**calcium ion binding**						
Zm00001eb230910	CDPK4	Calcium dependent protein kinase 4	0.11	NDE	-1.01	Down
Zm00001eb324690	CDPK20	Calcium dependent protein kinase 20	0.34	NDE	-1.25	Down
Zm00001eb135650	CML5	Calcium-binding protein 5	3.93	Up	-0.48	NDE
Zm00001eb352340	CML9	Calcium-binding protein 9	-	-	1.20	Up
Zm00001eb125050	CML16	Calcium-binding protein 16	-0.62	NDE	-1.01	Down
Zm00001eb395260	CML27	Calcium-binding protein 27	-0.01	NDE	-1.50	Down
Zm00001eb330780	CML32	Calcium-binding protein 32	3.14	Up	0.18	NDE
Zm00001eb176050	CML48	Calcium-binding protein 48	-1.49	Down	-1.31	Down
Zm00001eb264170	CBP	Calmodulin binding protein	-3.22	Down	-1.24	Down
Zm00001eb341260	RBOHB	Respiratory burst oxidase homolog protein B	6.06	Up	1.06	Up
Zm00001eb197890	RBOHD	respiratory burst oxidase homolog protein D	-0.97	NDE	-1.18	Down
**MAPK signaling pathway**						
Zm00001eb054290	MKKK1	Mitogen-activated protein kinase kinase kinase 1	-0.47	NDE	-0.99	NDE
Zm00001eb294610	MKKK17	Mitogen-activated protein kinase kinase kinase 17	0.83	NDE	-1.08	Down
Zm00001eb397190	MPK1	Mitogen-activated protein kinase 1	-0.12	NDE	-1.08	Down
Zm00001eb376640	MPK2	Mitogen-activated protein kinase 2	0.37	NDE	0.36	NDE
Zm00001eb013100	MPK3	Mitogen-activated protein kinase 3	0.12	NDE	-1.25	Down
Zm00001eb226770	MPK4	Mitogen-activated protein kinase 4	-0.15	NDE	-0.93	NDE
Zm00001eb199530	WRKY2	WRKY-transcription factor 2	-1.70	Down	0.26	NDE
Zm00001eb149300	WRKY24	WRKY-transcription factor 24	-0.95	NDE	-1.84	Down
Zm00001eb203940	WRKY27	WRKY-transcription factor 27	-1.02	Down	-1.7	Down
Zm00001eb169460	WRKY75	WRKY-transcription factor 75	1.26	Up	-1.93	Down
**ABA signaling pathway**						
Zm00001eb013780	PYL4	Abscisic acid receptor PYL 4	-0.24	NDE	-1.05	Down
Zm00001eb390480	PYL10	Abscisic acid receptor PYL 10	-2.25	Down	0.26	NDE
Zm00001eb160470	PP2C6	Protein phosphatase 2C 6	-1.29	Down	0.26	NDE
Zm00001eb419870	PP2C37	Protein phosphatase 2C 37	-1.03	Down	1.37	Up
Zm00001eb023880	SnRK2.3	SnRK2 serine threonine protein kinase 3	-0.43	NDE	1.34	Up
Zm00001eb051510	SnRK2.9	SnRK2 serine threonine protein kinase 9	-1.46	Down	0.69	NDE
Zm00001eb289080	bZIP12	bZIP transcription factor 12	-1.34	Down	-0.14	NDE
Zm00001eb366900	bZIP75	bZIP transcription factor 75	0.41	NDE	-1.20	Down
**Cation transporters**						

Zm00001eb292010	CNGC4	Cyclic nucleotide-gated ion channel 4	6.67	Up	-0.24	NDE
Zm00001eb068710	CNGC9	Cyclic nucleotide-gated ion channel 9	-1.26	Down	-0.54	NDE
Zm00001eb158420	AKT1	Potassium channel 1	-5.55	Down	-2.31	Down
Zm00001eb000570	AHA9	ATPase 9, plasma membrane-type	-3.06	Down	-0.16	NDE
Zm00001eb130310	HKT1	Sodium transporter 1	-3.20	Down	2.48	Up
Zm00001eb360320	TPC1	Two pore calcium channel protein 1	-0.23	NDE	-0.31	NDE
Zm00001eb387010	AVP5	Vacuolar-type H^+ pyrophosphatase 5	1.59	Up	-0.05	NDE
Zm00001eb067920	CAX3	Vacuolar cation/proton exchanger 3	1.58	Up	0.14	NDE
Zm00001eb165870	NHX2	Sodium/hydrogen exchanger 2	1.69	Up	-0.46	NDE
Zm00001eb381570	NHX4	Sodium/hydrogen exchanger 4	2.35	Up	0.83	NDE
**Peroxisome**						
Zm00001eb002510	CAT2	Catalase isozyme 2	-0.98	NDE	0.17	NDE
Zm00001eb226600	SOD15	Superoxide dismutase 15	-1.47	Down	0.67	NDE
Zm00001eb330020	SOD2	Superoxide dismutase 2	-1.37	Down	0.41	NDE
Zm00001eb140320	POD12	Peroxidase 12	-2.22	Down	-1.74	Down
**Other transcription factors**						
Zm00001eb157270	ARF2	Auxin response factor 2	-2.52	Down	0.42	NDE
Zm00001eb082150	ARF9	Auxin response factor 9	-0.2	NDE	-0.24	NDE
Zm00001eb067270	ARF11	Auxin response factor 11	0.81	NDE	0.81	NDE
Zm00001eb082180	bHLH18	Transcription factor bHLH 18	-1.87	Down	1.20	Up
Zm00001eb011370	bHLH30	Transcription factor bHLH 30	-7.21	Down	1.79	Up
Zm00001eb307550	ERF1B	Ethylene-responsive transcription factor 1B	-0.31	NDE	-0.53	NDE
Zm00001eb176840	NAC30	NAC domain-containing protein 30	4.68	Up	2.52	Up
Zm00001eb320820	NAC45	NAC domain-containing protein 45	-3.48	Down	1.33	Up
Zm00001eb024300	NAC86	NAC domain-containing protein 86	-4.87	Down	-1.71	Down

Note: 'Up' indicates an increase ; 'Down' indicates down-regulation ; 'NDE' indicated no differential expression.

Supplementary Table S4. m^{6}A-IP/input RNA quality inspection

Sample name	Concentration(ng/ul)	Volume(ul)	Gross(ug)	RINe value
CK-1	180	140	25.2	9.5
CK-2	176	139	24.9	9.3
CK-3	185	145	25.6	9.6
ST-1	202	139	28.1	9.1
ST-2	197	137	27.3	8.9
ST-3	203	142	28.9	9.3

Supplementary Table S5. List of primer pairs used in qRT-PCR

Primer Name	F	R
RBOHB	GGTTCTTTTTCCTGTTTGCC	CCACCCTTCAGTTCCCTTT
AKT1	CACGAAAAGCACACAGGAA	GAGATGACGCCGAAAAGAG
HKT1	CGTGCTGGGCTACATCTTGG	CATCCCCTCGTTGGTTGG
POD12	CAGGACGCCTTCTTCCACCAGTT	TCGATGACGACGGACGACCAC
bHLH18	ATGGATGGCAAGGGTGTGG	CAAAGTGCAAGCCTGGAATGAC
NAC86	AGAGGGTGATGGCGTCTTTTTT	GGCAGGAACGACTTGTCTGGTAG
